# Exploring 12-Year trends in childhood obesity prevalence for the Republic of Ireland – a national study using survey data from 2002 and 2014

**DOI:** 10.12688/hrbopenres.12988.1

**Published:** 2021-01-06

**Authors:** Seán R. Millar, Mairead Harding, Laura E. McCarthy, Virginia Kelleher, Janas M. Harrington, Patrice James, Helen Whelton, Ivan J. Perry

**Affiliations:** 1 School of Public Health, University College Cork, Cork, Co Cork, Ireland; 2 Oral Health Services Research Centre, University College Cork, Cork, Co Cork, Ireland; 3 Cork University Dental School and Hospital, University College Cork, Cork, Co Cork, Ireland; 4 Cork Kerry Community Healthcare, Health Service Executive, Cork, Co Cork, Ireland; 5 College of Medicine and Health, University College Cork, Cork, Co Cork, Ireland

**Keywords:** Overweight, Obesity, Children, Cohort, Economic disadvantage, Ireland

## Abstract

**Background:** The prevalence of overweight and obesity among children may have reached a plateau in some developed countries, including Ireland. The aim of this study was to examine 12-year trends in the prevalence of overweight and obesity among primary-school-aged children in the Republic of Ireland between 2002 and 2014.

**Methods: **Two large-scale oral health cross-sectional surveys of primary-school-aged children aged 4–13 years were conducted 12 years apart in 2002 (n=14,055; response rate=68%) and 2014 (n=5,223; response rate=67%). Both surveys included standardised and calibrated height and weight measures. Ownership of a means-tested medical card determined economic status. Standard International Obesity Task Force criteria were applied to determine the prevalence of overweight and obesity.

**Results:** The overall prevalence of overweight/obesity among 4–13-year-olds significantly decreased from 26% (95% CI: 25.1%–26.5%) in 2002 to 24% (95% CI: 22.4%–24.7%) in 2014. Among boys and girls aged 4–13 years, the significant decrease was from 23% (95% CI: 22.1%–24.1%) to 20% (95% CI: 18.9%–22.0 and 28% (95% CI: 27.4%–29.5%) to 27% (95% CI: 25.0%–28.4%), respectively. Among 5-year-old children, the overall prevalence of overweight and obesity significantly decreased from 25% (95% CI: 23.7%–26.2%) to 22% (95% CI: 19.9%–23.4%). In 2002, the estimated prevalence of overweight/obesity was similar in children with and without medical cards, whereas in 2014, overall prevalence was higher in those with medical cards.

**Conclusions:** Results suggest a fall in the prevalence of overweight/obesity between 2002 and 2014, and may suggest a favourable cohort effect. Despite this fall, the overall prevalence remains high and socioeconomic disparities have increased.

## Introduction

Childhood obesity remains a global public health priority. In the Republic of Ireland there was a sharp increase in the prevalence of overweight and obesity between the mid-20 ^th^ century and the start of the 21 ^st^ century. During this period, the average weight of 14-year-olds increased by 65% among boys and 48% among girls, with most of the increase occurring between 1970 and 2002 ^1^. This trend has been mirrored in most developed and developing countries over the last 30 years ^2^.

A high body mass index (BMI) in childhood is related to adverse outcomes in adult life such as a greater risk of morbidity ^3^, including some cancers ^4^ and type 2 diabetes ^5^. It is also associated with a greater likelihood of premature death ^6, 7^. In addition, excess weight in childhood is positively related to lower socioeconomic status ^8^ and inversely associated with psychological well-being ^9– 12^. While the personal and social costs related to childhood overweight and obesity are incalculable, financial costs should also be considered. A recent study, conducted in 2017, estimated the total lifetime cost of childhood obesity to be €4.6 billion for the Republic of Ireland, with 21% of costs being direct healthcare costs and more than two-thirds (79%) being indirect costs due to absenteeism, premature mortality and lifetime income losses ^13^. This research also suggested that a 1% and 5% reduction in population mean childhood BMI would be associated with a €270 million and €1.1 billion reduction in projected lifetime costs attributable to childhood overweight/obesity in Ireland.

Between 2002 and 2014, 14 nationally or regionally representative studies were carried out in Ireland to determine childhood obesity prevalence estimates. In a systematic review of these studies there was some evidence of a plateau in childhood overweight and obesity ^14^. However, this apparent plateau was based on studies which were heterogeneous in their methods in terms of whether sampling frames were national or regional, in addition to varying response rates ^15^.

In a large national oral health survey of over 14,000 children conducted in 2002, 26% of boys and girls in primary school in the Republic of Ireland were overweight or obese ^16^. This survey also found that prevalence estimates did not vary greatly by economic status as defined on the basis of access to a medical card. In the current research, we have replicated the methods of the 2002 oral health survey in a sample of children recruited in 2014. The aim of this research was to estimate the prevalence of overweight and obesity among children in the Republic of Ireland (including variation by economic status), drawing on data from over 5,000 children aged 4–13 years, and to compare findings with results from the 2002 survey.

## Methods

### Sample

The methods used in the 2002 study have been reported elsewhere ^16^. Briefly, the sample was a stratified randomised cluster sample, with the school as the primary sampling unit. Schools were categorised according to health board region (Community Care Area), school size (small, medium or large) and, for oral health reasons, whether they were located in a fluoridated or non-fluoridated area. Within each Community Care/Dental Area, schools were randomly selected to ensure a balance for fluoridation status (where appropriate) and for size of the school. A list of all children in Junior Infants (5-year-olds) and in 6 ^th^ class (12-year-olds) was obtained from selected schools that consented to participate.

The selection of children was on the basis of age, sex, geographical location of the school attended and whether the school was located in a fluoridated or non-fluoridated area ^17^. The required number of children from each class (age group) was selected randomly and consent forms were issued only to those children selected. Informed consent was obtained from the parents/guardians of participating children and assent from the 12-year-old children ^17^. The sample of schools was prepared using stratified cluster random sampling. A list of primary schools in Dublin, Cork and Kerry in SY 2011–2012) formed the sampling frame for the study. Schools for children with special educational needs were not included in the sampling frame ^17^ The final sample included 14,055 children attending primary schools in Dublin, Cork and Kerry; We have replicated the methods of the dental health survey from 2002 in a sample of 5,223 children recruited in the 2014 study period (November 2013 to May 2014) ^17^. The Clinical Research Ethics Committee of the Cork Teaching Hospitals approved both oral health studies (National Survey of Children's Dental Health approved on 02/10/2001, FACCT (ECM 5 (2) 07/05/13).

### Measurement of height, weight and socioeconomic status

In the 2002 survey, height was measured using a Leicester height/length stadiometer (CMS Weighting Equipment, London, UK). Weights were measured using a Soehnle 7403 Mediscale (Soehnle Professional GmbH & Co, Murrhardt, Germany). For the 2014 survey, height was measured using a Seca 213 portable stadiometer (Seca, Birmingham, UK). Weight was measured using Tanita WB 100SMA weighing scales (Tanita Corporation, IL, USA). For both surveys, the weight scales were calibrated using 75kg calibration weights prior to the commencement of the study. BMI was calculated as weight divided by the square of height. Standard International Obesity Task Force (IOTF) criteria ^18^ were applied to determine prevalence estimates. Weight and height measurements were taken by researchers who were thoroughly trained according to the study research protocols.

Socioeconomic status was measured by medical card status (means-tested public sector healthcare). Anyone over the age of 16 years who is ordinarily resident in the Republic of Ireland can apply for a Medical Card (which entitles the holder to a range of free health services) or a GP visit card (which entitles the holder to visit the family doctor free-of-charge) ^19^. People qualify for these cards by being means-tested; weekly income must be below an income threshold. Children whose parents had a Medical Card or GP visit card were categorised as being economically disadvantaged; children whose parents did not have a Medical Card/GP card were categorised as being non-economically disadvantaged. 

### Data management and analysis

Data from the 2002 and 2014 surveys were recorded, cleaned and coded by the Oral Health Services Research Centre. Data analysis was conducted using SAS Version 9.4 (SAS Institute Inc., Cary, NC, USA). Two-sample t-tests between proportions were performed to determine statistical differences. Data are presented as proportions and 95% confidence intervals using binomial approximation. For all analyses, a *p* value (two-tailed) of less than 0.05 was considered to indicate statistical significance.

## Results

The overall response rates for children in the 2002 and 2014 surveys were 68% and 67%, respectively. The prevalence of childhood overweight and obesity for the years 2002 and 2014, according to the IOTF classification, are presented by age and sex in Table 1 ^20^. In 2014, among children aged 4–13 years, one-in-five boys (20%) and more than one-in-four girls (27%) were either overweight or obese. One-in-25 boys (4%) and approximately one-in-14 girls (7%) were obese. The prevalence of overweight was also higher in girls (20%) than boys (16%).

**Table 1.** Prevalence of overweight and obesity among primary school children in the Republic of Ireland in 2002 and 2014.

	2002	2014	Change in prevalence 2002 and 2014
**4–13-year-olds**			
**Overweight**			
Boys	17% (16.4% to 18.2%)	16% (14.7% to 17.6%)	-1%
Girls	21% (20.3% to 22.2%)	20% (18.6% to 21.7%)	-1%
Total	19% (18.6% to 19.9%)	18% (17.1% to 19.2%)	-1% ^*^
**Obese**			
Boys	6% (5.3% to 6.3%)	4% (3.5% to 5.1%)	-2% ^*^
Girls	7% (6.6% to 7.8%)	7% (5.6% to 7.5%)	0%
Total	6% (6.1% to 6.9%)	5% (4.8% to 6.0%)	-1% ^*^
**Overweight and obese**			
Boys	23% (22.1% to 24.1%)	20% (18.9% to 22.0%)	-3% ^*^
Girls	28% (27.4% to 29.5%)	27% (25.0% to 28.4%)	-1% ^*^
Total	26% (25.1% to 26.5%)	24% (22.4% to 24.7%)	-2% ^*^
**5-year-olds**			
**Overweight**			
Boys	17% (15.3% to 18.3%)	13% (11.2% to 15.3%)	-4% ^*^
Girls	22% (20.4% to 23.8%)	20% (17.4% to 22.3%)	-2%
Total	19% (18.2% to 20.4%)	16% (14.9% to 18.1%)	-3% ^*^
**Obese**			
Boys	5% (3.9% to 5.6%)	4% (3.0% to 5.4%)	-1%
Girls	7% (5.6% to 7.6%)	6% (4.7% to 7.6%)	-1%
Total	6% (5.0% to 6.3%)	5% (4.2% to 6.1%)	-1%
**Overweight and obese**			
Boys	22% (19.9% to 23.1%)	17% (15.1% to 19.6%)	-5% ^*^
Girls	29% (26.8% to 30.5%)	26% (23.3% to 28.7%)	-3%
Total	25% (23.7% to 26.2%)	22% (19.9% to 23.4%)	-3% ^*^
**12-year-olds**			
**Overweight**			
Boys	19% (17.2% to 21.5%)	18% (15.4% to 20.6%)	-1%
Girls	19% (16.9% to 21.0%)	21% (18.5% to 24.2%)	2%
Total	19% (17.7% to 20.6%)	20% (17.7% to 21.6%)	1%
**Obese**			
Boys	6% (4.6% to 7.2%)	4% (2.8% to 5.5%)	-2% ^*^
Girls	6% (5.0% to 7.5%)	7% (5.1% to 8.6%)	1%
Total	6% (5.2% to 7.0%)	5% (4.4% to 6.6%)	-1%
**Overweight and obese**			
Boys	25% (22.9% to 27.6%)	22% (19.4% to 25.0%)	-3% ^*^
Girls	25% (22.9% to 27.5%)	28% (25.1% to 31.4%)	3% ^*^
Total	25% (23.6% to 26.8%)	25% (23.1% to 27.3%)	0%

^*^statistically significant

Between 2002 and 2014, among children aged 4–13 years, there were statistically significant decreases in the prevalence of overweight from 19% (95% CI: 18.6%–19.9%) to 18% (95% CI: 17.1%–19.2%), of obesity from 6% (95% CI: 6.1%–6.9%) to 5% (95% CI: 4.8%–6.0%) and of overweight and obesity combined from 26% (95% CI: 25.1%–26.5%) to 24% (95% CI: 22.4%–24.7%). Among boys aged 4–13 years, there were significant decreases in both the prevalence of obesity from 6% (95% CI: 5.3%–6.3%) to 4% (95% CI: 3.5%–5.1%) and of overweight and obesity combined from 23% (95% CI: 22.1%–24.1%) to 20% (95% CI: 18.9%–22.0%). Among girls aged 4–13 years, a significant decrease was only seen in the prevalence of overweight and obesity combined from 28% (95% CI: 27.4%–29.5%) to 27% (95% CI: 25.0%–28.4%).

When only 5-year-olds were analysed, there was a statistically significant decrease in the prevalence of overweight from 19% (95% CI: 18.2%–20.4%) to 16% (95% CI: 14.9%–18.1%) and in the prevalence of overweight and obesity combined from 25% (95% CI: 23.7%–26.2%) to 22% (95% CI: 19.9%–23.4%) over the 12 years. Significant decreases were also observed among boys aged 5 years in the prevalence of overweight from 17% (95% CI: 15.3%–18.3%) to 13% (95% CI: 11.2%–15.3%) and in the prevalence of overweight and obesity combined from 22% (95% CI: 19.9%–23.1%) to 17% (95% CI: 15.1%–19.6%). Among 12-year-olds, while overall there were no statistically significant changes in prevalence between 2002 and 2014, among girls there was a significant increase in the prevalence of overweight and obesity combined from 25% (95% CI: 22.9%–27.5%) to 28% (95% CI: 25.1%–31.4%). Among boys aged 12 years, there were significant decreases in the prevalence of obesity from 6% (95% CI: 4.6%–7.2%) to 4% (95% CI: 2.8%–5.5%) and of overweight and obesity combined from 25% (95% CI: 22.9%–27.6%) to 22% (95% CI: 19.4%–25.0%).

In 2002, 23% (n=3,244) of the sample were characterised as economically disadvantaged compared to 35% (n=1,831) in 2014. In the 2002 survey, children who were economically disadvantaged compared to those who were non-economically disadvantaged had only slightly higher rates of obesity (7.6% vs. 6.2%) and of overweight and obesity combined (26.1% vs. 25.7%) ( Table 2) ^20^. However, over the 12-year period this gap increased, with higher rates of overweight (20.5% vs. 16.6%) and obesity (7.6% vs. 4.2%) among children who were economically disadvantaged. The prevalence of overweight and obesity combined was 28.0% in 2014 among children who were from economically disadvantaged families compared to 20.9% among children who were non-economically disadvantaged.

**Table 2.** Prevalence of overweight and obesity among primary school children in the Republic of Ireland in 2002 and 2014 by Economic Disadvantage (ED) status.

	2002	2014	Change in prevalence 2002 and 2014
**4–13-year-olds**			
**Overweight**			
Not ED	19.5% (2094)	16.6% (549)	-2.9%
ED	18.5% (600)	20.5% (372)	2.0%
Difference between ED and not ED	**-1.10%**	**3.81%**	**4.91%**
**Obese**			
Not ED	6.2% (660)	4.2% (139)	-2.0%
ED	7.6% (247)	7.6% (138)	0.0%
Difference between ED and not ED	**1.50%**	**3.38%**	**1.88%**
**Overweight or obese**			
Not ED	25.7% (2754)	20.9% (688)	-4.8%
ED	26.1% (847)	28.0% (510)	1.9%
Difference between ED and not ED	**0.40%**	**7.19%**	**6.79%**
**5-year-olds**			
**Overweight**			
Not ED	20.0% (740)	15.6% (216)	-4.4%
ED	16.7% (170)	17.8% (120)	1.1%
Difference between ED and not ED	**-3.30%**	**2.17%**	**5.47%**
**Obese**			
Not ED	5.5% (202)	4.2% (58)	-1.3%
ED	6.1% (62)	7.3% (49)	1.2%
Difference between ED and not ED	**0.60%**	**3.07%**	**2.47%**
**Overweight or obese**			
Not ED	25.5% (942)	19.8% (274)	-5.7%
ED	22.8% (232)	25.0% (169)	2.2%
Difference between ED and not ED	**-2.70%**	**5.24%**	**7.94%**
**12-year-olds**			
**Overweight**			
Not ED	18.3% (386)	18.2% (197)	-0.1%
ED	21.9% (129)	22.3% (116)	0.4%
Difference between ED and not ED	**3.70%**	**4.10%**	**0.40%**
**Obese**			
Not ED	5.6% (119)	4.0% (43)	-1.6%
ED	7.7% (45)	8.5% (44)	0.8%
Difference between ED and not ED	**2.00%**	**4.49%**	**2.49%**
**Overweight or obese**			
Not ED	23.9% (505)	22.2% (240)	-1.7%
ED	29.6% (174)	30.8% (160)	1.2%
Difference between ED and not ED	**5.70%**	**8.59%**	**2.89%**

## Discussion

This paper presents data from two large national studies of Irish children aged 4–13 years who were examined in 2002 and 2014 by trained oral health survey teams, coordinated by the Oral Health Services Research Centre, University College Cork, using standardised methods. In this 12-year period there has been evidence of a slight fall in the overall prevalence of overweight and obesity combined, with a statistically significant decrease of 2% among children aged 4–13 years. It should be noted that some of the differences observed may be due to the age distribution of the sample. The dental survey targeted Junior Infants and 6 ^th^ class students, hence the majority of the sample are 5-year-olds and 12-year-olds, with a clustering of ages around these target years. Therefore, we focused on age-specific trends. There was no change in the prevalence of overweight and obesity among 12-year-olds. However, there was a significant decrease of 3% in the prevalence of overweight and obesity among 5 year-olds, which suggests a possible favourable cohort effect.

The findings from this study are consistent with previous evidence suggesting that childhood obesity rates have stabilised in the Republic of Ireland ^14, 15, 21^, in addition to some countries in Europe and in Australia, Japan and the USA ^22^; they may be decreasing in other countries such as Sweden ^23^, Denmark ^24^ and Switzerland ^25^. However, while there may be a favourable trend overall, the prevalence gap between economically disadvantaged and non-economically disadvantaged children in the Republic of Ireland is cause for concern. It should be noted that the 2002 survey was carried out at time of affluence in Ireland at the height of the Celtic Tiger years, whereas the 2014 study was held against the backdrop of the 2008 recession. Nevertheless, the socioeconomic gap in childhood obesity is also evident in the United Kingdom ^26, 27^, while widening socioeconomic disparities in childhood overweight have been reported in Sweden ^28^ and other countries across Europe ^29^.

The Irish research evidence suggests that socioeconomic inequalities create living conditions that are damaging to health, most particularly for poor and vulnerable members of society. In this context, gathering best evidence on tackling health inequalities and reconfiguring what needs to be done has never been more important ^30^. From a public health perspective, these findings suggest that targeted strategies to reduce the impacts of socioeconomic inequalities are needed. Children attending disadvantaged schools deserve special attention, especially as they approach adolescence, and health promotion policies should target the obesogenic environment they are exposed to ^31^. However, there is a lack of evidence of the health inequality impact of existing policies and interventions in the Irish context ^30^.

The Healthy Weight for Ireland Obesity Policy and Action Plan 2016–2025 outlines a 5-year target of reducing the prevalence of overweight and obesity combined amongst children in the Republic of Ireland by 0.5% per annum. It also aims to reduce the gap in obesity levels between the highest and lowest socioeconomic groups by 10% ^32^. If these targets were achieved, we would expect to see a further fall in the prevalence of childhood overweight and obesity from 24% to 21% by 2020. Actions that have been implemented, or which are soon to be realised, include a tax on sugar-sweetened beverages, a formal review of the advertising of energy dense food and drinks to children, the expansion and wide scale implementation of effective school initiatives/interventions on healthy eating and physical activity in schools as well as the introduction of national food standards for primary schools.

The inclusion of obesity prevention and care as part of primary care (including implementation of an appropriate child growth monitoring system) is another important development within Ireland ^32^. As previously noted, to date, childhood obesity prevalence rates in Ireland have been assessed using studies that are heterogeneous in their methods with regard to national or regional sampling frames. Information from general practices could supply an annual source of objectively measured child BMI data that might provide accurate and reliable population-based data for the routine monitoring of childhood overweight and obesity in order to examine patterns and trends. These data could also be used for assessing the efficacy of the obesity prevention health interventions outlined above. Data collected could also include information on utilisation of healthcare services (both primary care and hospital services) and illness-related productivity loss and absenteeism from work and school for conducting cost-of-illness studies and other related health economic analysis ^13^. In particular, data from general practices could provide an annual source of BMI/health-related data for children who are under 5 years of age – which has been lacking.

### Strengths and limitations

A major strength of this study is that response rates were very similar in the 2002 and 2014 surveys (68% and 67%, respectively) and that the socioeconomic backgrounds of study participants broadly reflect the national data. In 2002, 1.2 million people (30% of the population) were covered by medical cards ; in 2014, 1.8 million people (39% of the population) were covered by medical cards and 142,668 people (3% of the population) were in receipt of GP visit cards ^19^. A potential limitation of this research is that the 2014 survey included a smaller number of schools and participants than the 2002 survey and the sample was recruited from Counties Cork and Kerry and six community care areas in Dublin. However, considering that both studies used the same sampling methods, this is unlikely to result in any socio-demographic differences between the two study samples. When the same geographical areas (Cork, Kerry and Dublin) were compared in 2002 and 2014, the direction and magnitude of the effect was the same. Results are therefore sensitive at a sub-population level.

## Conclusions

The findings from this research are consistent with stabilisation, and a possible declining trend, in the prevalence of overweight and obesity among Irish children aged 4–13 years between 2002 and 2014. The trend was most marked and statistically significant within the subgroup of five-year-old children, raising the possibility of a favourable birth cohort effect. However, evidence for an increase in social inequality among overweight and obese children is important and should be a key public health policy issue.

## Data availability

### Underlying data

Zenodo: Exploring 12-Year Trends in Childhood Obesity Prevalence for the Republic of Ireland – A National Study Using Survey Data from 2002 and 2014. http://doi.org/10.5281/zenodo.4265394 ^20^

This project contains the following underlying data:

- Excel File BMI data 2002 and 2014 (Aggregate data for 2002 and 2014, BMI, Age, Medical card (MC, economic disadvantage)).
- Excel File FACCT Ireland 2002 and 2014 (Individual data, Year of survey, Height (cm), Weight (kg), calculated BMI, Age, Medical card (MC, economic disadvantage)).

Data are available under the terms of the Creative Commons Attribution 4.0 International license (CC-BY 4.0).
