# Supplementary material for: Exploring 12-Year trends in childhood obesity prevalence for the Republic of Ireland – a national study using survey data from 2002 and 2014
Source: HRB Open Res. 2021 Jan 6;4:3. [Version 1] doi: 10.12688/hrbopenres.12988.1 (PMC9334843; doi:10.12688/hrbopenres.12988.1)
Supplement: Peer review history

# Peer review: Reviewer response for version 1

**DOI:** 10.21956/hrbopenres.14074.r32534

**Published:** 2022-07-28

**Authors:** Frank Sullivan, Katrin Metsis

**Affiliations:** 1 Population and Behavioural Sciences, School of Medicine, University of St Andrews, St Andrews, UK; 2 University of St Andrews, St Andrews, UK

The aim of the study by Millar *et al*. 2021 is to examine 12-year trends of overweight and obesity among primary-school-aged children in the Republic of Ireland. The study uses data from the large oral health surveys completed in 2002 and 2014. The particular interest is the possible existence of a plateau in childhood overweight and obesity, and the variation of overweight and obesity by the economic status. Overall, this study is very timely and beneficial because there is not much measured height and weight data available. We would like to make the following comments.

- The aim of the study is stated differently in the Abstract and Introduction.

*The aim of this study was to examine 12-year trends in the prevalence of overweight and obesity among primary-school-aged children in the Republic of Ireland between 2002 and 2014.*

*V*

*The aim of this research was to estimate the prevalence of overweight and obesity among children in the Republic of Ireland (including variation by economic status), drawing on data from over 5,000 children aged 4–13 years, and to compare findings with results from the 2002 survey.*

**Suggestion: While neither of the stated aims is incorrect, it would be beneficial to state that in coherent way throughout the study**.

- Reference to the “large-scale oral health survey” is confusing. The study which used data from the 2002 survey, refers to the survey as “the North South Survey of Children’s Height, Weight and Body Mass Index, 2002” (Whelton *et al*. 2007). Because of the reference to the “large oral health survey” the impression is that weight and height measurements were a part of the oral health survey.

**Suggestion: It would be desirable to include the explicit references to the 2002 and 2014 surveys within the article**.

- Measurement of the BMI. The measurement of the BMI is complex in children and there is no universally agreed criteria for doing this.

Question: the current study refers to the IOTF criteria in the 2011 publication by Monasta *et al.* (2011). The study by Monasta *et al. *(2011) compares the IOTF standard to the WHO standard by using the data for 24–60-month-old children. The population of the current study is 4- to 13-year-olds. For example, study by Whelton *et al*. (2007) has referred to the IOTF 2002 publication, this uses cut points as proposed by Cole *et al. *2000. ^1^

**Suggestion: Clarify the criteria used at different ages.**

- Use of the medical card to denote socioecomic status may reduce generalisability to other health systems.  A description of how the cards are provided and the extent of uptake in the target group may allow comparison with other health and social care systems.
- It was interesting that study by Whelton *et al.* (2007) which used 2002 data did not find differences by the socioeconomic disadvantage as measured by the ownership of the Medical Card. A good possible explanation is provided for this, the RI had a period of affluence during the 2002 survey which seemingly benefitted also disadvantaged population groups.

Question: In the study by Whelton *et al.* (2007), the differences by socioeconomic disadvantage were examined separately for boys and girls by age. Current study finds differences in the boys and girls overweight and obesity prevalence, however, the differences by disadvantage are examined by the age only. Did you try analysis by gender, and if yes, were the results different for boys and girls?

- A Table 1 comparing the composition of the 2 samples wrt age, sex and socioeconomic status would be useful.

Table titles: Table 1 add “by age and gender”; Table 2 add “by age”.

- Include “overweight” in the title. Also using the term trends may be misleading in a repeated cross-sectional study e.g.  patterns or measures or a comparison…..
- Good discussion of strengths and limitations: similar response rates for 2002 and 2014 survey, and similarity of socioeconomic background of study respondents compared to the national data. Also, the potential limitation of the overall smaller number of respondents and schools in the 2014 survey is highlighted. This limitation is well discussed.

Is the work clearly and accurately presented and does it cite the current literature?

Partly

If applicable, is the statistical analysis and its interpretation appropriate?

Partly

Are all the source data underlying the results available to ensure full reproducibility?

Yes

Is the study design appropriate and is the work technically sound?

Partly

Are the conclusions drawn adequately supported by the results?

Yes

Are sufficient details of methods and analysis provided to allow replication by others?

Partly

Reviewer Expertise:

Clinical epidemiology, adolescent health.

We confirm that we have read this submission and believe that we have an appropriate level of expertise to confirm that it is of an acceptable scientific standard.

## References

[1] : Establishing a standard definition for child overweight and obesity worldwide: international survey. *BMJ* .2000;320(7244) : 10.1136/bmj.320.7244.1240 1240-3 10.1136/bmj.320.7244.1240 10797032PMC27365

# Peer review: Reviewer response for version 1

**DOI:** 10.21956/hrbopenres.14074.r29079

**Published:** 2021-04-06

**Authors:** Duarte Henriques-Neto

**Affiliations:** 1 CIPER, Faculty of Human Motricity, Universidade De Lisboa, Lisboa, Portugal

This manuscript aimed to estimate the prevalence of overweight and obesity among children in the Republic of Ireland in 2014 and compare with data from 2002. Also, the variation between 2002 and 2014 was stratified by economic status. For doing so the authors used the BMI categories. Main findings suggest that the overall prevalence of overweight/obesity among 4–13 year-olds significantly decreased between 2002 and 2014. It is a well-written manuscript and clear to read.

 I have only a few suggestions/remarks to do. When we study weight status or other fitness attributes, the maturity stage is an important variable to be accountable for, but at the same time, difficult to assess in epidemiologic studies, especially in this age range (10-13yrs). In fact, one of the major limitations of the study is the lack of adjustment to confounding variables (e.g. socioeconomic status, physical activity, nutrition, maturity), especially considering that these were two independent samples. This should be stated as a limitation.

 Also, the authors affirm using a two-sample t-tests between proportions, however it is not clear whether this was an independent sample t-test or a paired sample t-test. From what I understood it was an independent sample t-test because the sample is different, is that so?

 Overall, congratulations to the authors for this important work.

Is the work clearly and accurately presented and does it cite the current literature?

Yes

If applicable, is the statistical analysis and its interpretation appropriate?

Yes

Are all the source data underlying the results available to ensure full reproducibility?

Yes

Is the study design appropriate and is the work technically sound?

Yes

Are the conclusions drawn adequately supported by the results?

Yes

Are sufficient details of methods and analysis provided to allow replication by others?

Yes

Reviewer Expertise:

Physical Fitness; Physical Activity and Health in youth. Bone Health in young athletes.

I confirm that I have read this submission and believe that I have an appropriate level of expertise to confirm that it is of an acceptable scientific standard.

# Response

**Published:** 2021-05-25

**Authors:** Mairead Harding

**Affiliations:** UCC, Ireland

Thank You Duarte,

 We are very grateful to receive your feedback and compliment on the research completed.

 In our study we used an independent t-test.

 We will continue to research in this area and conduct further analysis with the availabler data.

 Kind regards

 Máiréad Harding
